# Comparison of *in vitro* and *in vivo* Toxicity of Bupivacaine in Musculoskeletal Applications

**DOI:** 10.3389/fpain.2021.723883

**Published:** 2021-08-20

**Authors:** Jasper G. Steverink, Susanna Piluso, Jos Malda, Jorrit-Jan Verlaan

**Affiliations:** ^1^Department of Orthopedics, University Medical Center Utrecht, Utrecht University, Utrecht, Netherlands; ^2^Regenerative Medicine Utrecht, Utrecht University, Utrecht, Netherlands; ^3^Department of Developmental BioEngineering, Technical Medical Centre, University of Twente, Enschede, Netherlands; ^4^Department of Clinical Sciences, Faculty of Veterinary Medicine, Utrecht University, Utrecht, Netherlands

**Keywords:** tissue, regeneration, bone, muscle, wound healing, orthopedic

## Abstract

The recent societal debate on opioid use in treating postoperative pain has sparked the development of long-acting, opioid-free analgesic alternatives, often using the amino-amide local anesthetic bupivacaine as active pharmaceutical ingredient. A potential application is musculoskeletal surgeries, as these interventions rank amongst the most painful overall. Current literature showed that bupivacaine induced dose-dependent myo-, chondro-, and neurotoxicity, as well as delayed osteogenesis and disturbed wound healing *in vitro*. These observations did not translate to animal and clinical research, where toxic phenomena were seldom reported. An exception was bupivacaine-induced chondrotoxicity, which can mainly occur during continuous joint infusion. To decrease opioid consumption and provide sustained pain relief following musculoskeletal surgery, new strategies incorporating high concentrations of bupivacaine in drug delivery carriers are currently being developed. Local toxicity of these high concentrations is an area of further research. This review appraises relevant *in vitro*, animal and clinical studies on musculoskeletal local toxicity of bupivacaine.

## Introduction

Musculoskeletal surgeries rank amongst the most painful surgical interventions overall (1). The debilitating effects of postoperative pain include delayed mobilization and complications, such as pneumonia and decubitus, which in turn can lead to increased hospital stay and re-admissions (2). Pain resulting from musculoskeletal surgery is usually treated with multimodal pain regimens in which opioids often fulfill an important role. Opioids have, however, well-known side effects such as nausea, respiratory depression and drowsiness, and can lead to dependence and addiction. Of all patients enrolling in opioid-abuse treatment programs, almost half were first exposed to opioids through prescription from their physician (3, 4). Currently, an estimated two million American citizens are addicted to prescription pain killers, such as opioids (5). The risks of dependence and abuse are especially relevant for patients undergoing musculoskeletal surgery (6). Despite administration of opioids, poorly controlled postoperative pain is reported by 75% of patients (2). Therefore, the interest in alternative pain treatments not displaying the systemic side effects of current opioid-based regimes is increasing. The use of local anesthetics (LAs) has the potential to accelerate postoperative recovery and reduce opioid consumption in musculoskeletal surgery. To this end, bupivacaine is especially popular as it displays the longest duration of action of all LAs (up to 8 h) (7). In comparison, the effects of lidocaine last up to 2 h in soft tissue (8). Because of its extensive clinical use and the highest potency compared to other long-acting amino-amide LAs such as ropivacaine and levobupivacaine, this review focuses on bupivacaine (9). However, 8 h of analgesia is likely insufficient for postoperative pain control and therefore novel formulations aim to further extend the duration of action of LAs. In fact, bupivacaine is the main LA that has been incorporated in recent opioid-free inventions for postsurgical pain relief (10–14).

Recent studies have expressed concerns regarding the *local* toxic effects of bupivacaine infiltration when used for musculoskeletal applications. This review aims to assess (i) the *in vitro, in vivo* and clinical effects of bupivacaine on various musculoskeletal tissues, cell types and relevant environments (including cartilage, bone, muscle, nerves, intervertebral disc, and surgical wounds) and (ii) and the clinical translatability and real-world relevance of these effects. Furthermore, recent approaches and developments to decrease the toxic profile and increase the duration of action of bupivacaine are discussed. Studies providing data on *in vitro*, animal model or clinical local toxicity of bupivacaine were manually selected. References of selected papers were checked for relevant literature. In addition, separate searches were performed for studies comparing free bupivacaine with novel formulations of bupivacaine (e.g., liposomal bupivacaine). Articles published in languages other than English and case reports were excluded from review. Studies describing systemic toxicity following administration of bupivacaine were outside the scope of this review. This literature review did not require an ethical board approval because no patients or patient data were involved in the review, and only publicly available data was used.

## Bupivacaine

**Figure 1:**
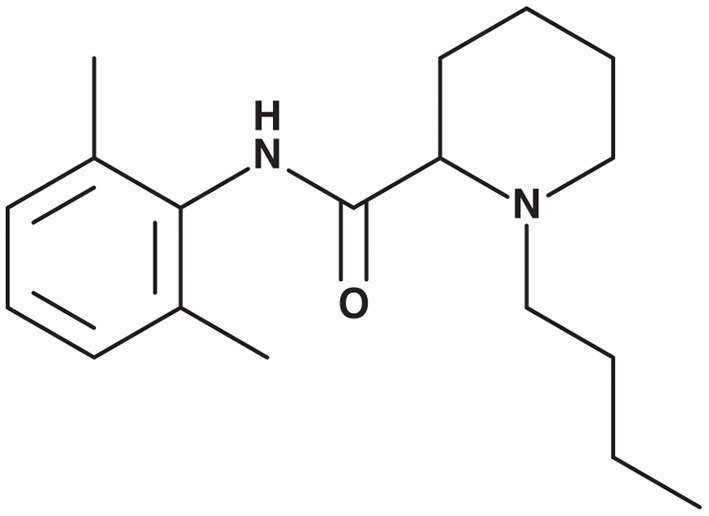
Bupivacaine structure formula.

After its discovery in 1957, bupivacaine (1-Butyl-N-(2,6-dimethylphenyl)-2-piperidinecarboxamide) (Figure 1) has become one of the most widely and frequently used LAs, and is listed as a World Health Organization Essential Drug (15, 16). Bupivacaine belongs to the class of amino-amide LAs, exerting their anesthetic action through binding to the intracellular portion of voltage-gated sodium channels, more specifically the alpha subunit. By inhibiting sodium influx into axons, depolarization and, therefore, pain signal transduction is inhibited (16). Examples of LA application in musculoskeletal surgery are local infiltration anesthesia (LIA) after total hip and total knee arthroplasty (THA and TKA, respectively). These local applications are associated with low rates of *systemic* toxicity and adverse effects, providing a favorable comparison with opioids (17–20). Besides therapeutic applications, LA infiltrations also serve as diagnostic tool in a variety of joint disorders, such as hip or shoulder osteoarthritis (21).

The class of amino-amide LAs has been derived from amino-ester LAs, such as cocaine. All amino-amide LAs have a similar molecular structure, with an aromatic lipophilic portion linked with an amide-containing intermediate chain to an amine hydrophilic portion (22). Bupivacaine has the longest duration of action (up to 8 h) of all amino-amide LAs due to its butyl group attached to the tertiary amine. The lipophilic butyl group allows for easy membrane-crossing resulting in high anesthetic potency and prolonged duration of action. In plasma, 95% of bupivacaine is protein-bound, mainly to albumin. Bupivacaine is metabolized in the liver by conjugation with glucuronic acid and excreted renally (23). Elimination half-life is ~2.7 h in adults and 8.1 h in neonates (24). The recommended maximum doses are 2 mg/kg body weight or a maximum of 400 mg per day. The dose can be increased to 2.5 mg/kg body weight with addition of epinephrine, by virtue of decreased systemic uptake resulting from local vasoconstriction (25). The left-isomer-only formulation of bupivacaine, levobupivacaine, is commercially available separately and has been associated with decreased systemic toxicity, predominantly cardiotoxicity (9). In *in vitro* studies, the concentration of bupivacaine is often reported in mM3 or uM. To help comparison between studies presented in this review, it should be noted that a clinically used concentration of 0.5% Bupivacaine HCl solution equals a molarity of 15.4 mM. Bupivacaine HCl solutions are registered for use in spinal, epidural, regional, or local infiltrative anesthesia. In all cases the solution is administered by injection.

## Effects of Bupivacaine on Skeletal Muscle

In musculoskeletal surgery, exposing osseous structure(s) often requires cutting through or releasing muscle tissue. Muscle recovery after surgery is of paramount importance, since it essential for wound healing, and facilitates quick mobilization and uneventful return to preoperative function.

It is known that LAs are myotoxic *in vitro*. Bupivacaine is reported to lead to 60–100% *in vitro* myocyte toxicity in concentrations >1 mM (i.e., in clinically used concentrations) (26). Previous studies have described histopathological changes at the injection site of LAs (27–30). Within min, hypercontraction of fiber bundles and myofibrils occurs. Within hours, a degenerative phase is observed, with disruption and condensation of myofilaments, lytic degeneration of the sarcoplasmic reticulum and mitochondria, and pathologic condensation of chromatin, which are signs of early necrosis and apoptosis. The sarcolemmal structure remains intact, potentially indicating that fiber degeneration occurs mainly intracellularly and does not affect the macroscopic structure of the muscle. This degenerative phase lasts for 24–48 h, after which phagocyte infiltration of the application site occurs. Debris is cleared without damage to basal laminae and satellite cells. As these cells remain largely intact, complete muscular regeneration can occur within 3–4 weeks.

It is hypothesized that the myotoxic effects of bupivacaine are induced by calcium influx in muscle cells. This is supported by the finding that bupivacaine in concentrations >>1 mM leads to activation of the Ca-release channel-ryanodine receptor in the sarcoplasmic reticulum (27, 31). Interestingly, levobupivacaine has been shown to have stronger effects on Ca-uptake in muscle cells compared with racemic bupivacaine, theoretically potentiating its myotoxicity (31, 32). Another factor that might play a key role in myotoxicity is pH. Indeed, the free base form of LAs reaching the sarcoplasmic reticulum is thought to be responsible for myocyte injury. At higher pH (i.e., closer to and beyond the drug's pKa), the free base fraction increases, potentiating bupivacaine's capacity to damage muscle (33).

**Figure 2:**
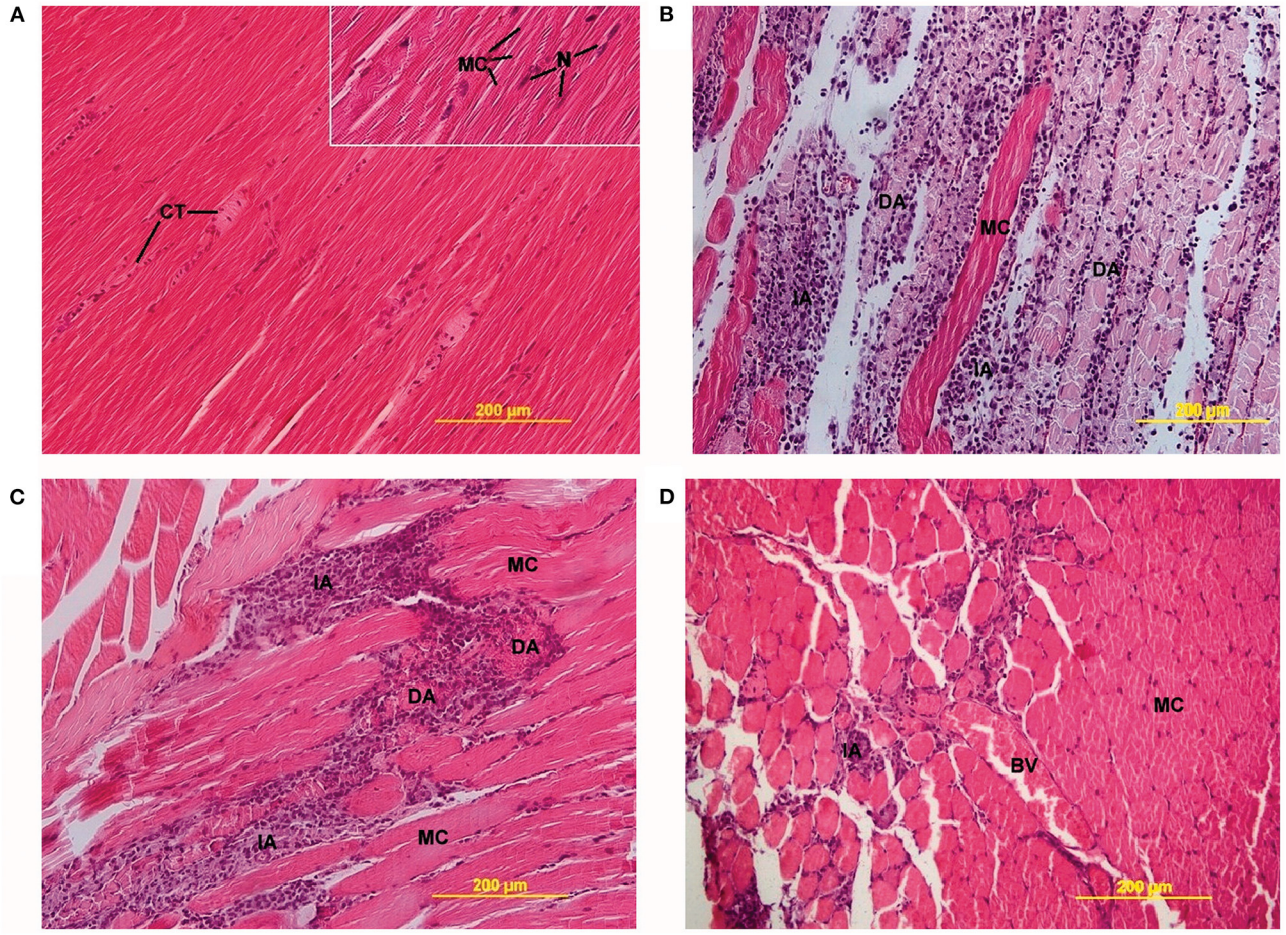
Light microscopy images of LA-induced myotoxicity. **(A)** Skeletal muscle 2 days after 0.9% saline injection, showing connective tissue (CT) between normal muscle fibers (MC) (20× magnification, insert 40×). **(B)** Skeletal muscle 2 days after 0.5% bupivacaine injection, showing degenerative (DA) and inflammatory areas (IA) alternated with MC (20× magnification). **(C)** Skeletal muscle 2 days after 0.5% ropivacaine injection, also displaying DA and IA (20× magnification). **(D)** Skeletal muscle 2 days after 0.5% levobupivacaine injection, showing incidental IA and blood vessels (BV) between MC (20× magnification). Öz Gergin et al. Comparison of the Myotoxic Effects of Levobupivacaine, Bupivacaine, and Ropivacaine: An Electron Microscopic Study. Ultrastructural Pathology, May 2015. Reprinted by permission of the publisher (Taylor & Francis Ltd.) (34).

Bupivacaine displays considerable myotoxicity *in vitro*, leading to apoptosis and (myo-)necrosis (Figure 2) (34–36). Bupivacaine-induced myotoxicity is dose and time dependent (37), with the myotoxic effects being reversible within 3 weeks (38). Animal models have been established to study muscle damage using bupivacaine as an agent to induce myotoxicity (39). A recent systematic review summarized the *in vivo* myotoxicity of bupivacaine and its effect on muscle recovery. Myotoxicity was defined as the presence of (a combination of) muscle weakness or paralysis, or enzymatic changes indicative of muscle damage (e.g., elevated creatine phosphokinase serum levels). Muscle tissue recovery time was defined as regeneration of muscle fibers, normalization of myofibil diameters, and resolution of inflammation and was doubled after LA administration (up to 30 days, vs. normal regeneration time of 14 days) (26, 40). Full recovery to preoperative function was reported in 21 studies, partial recovery in 17 studies and minimal recovery in four studies (26). Notably, myotoxicity also occurred after administration of liposomal bupivacaine (26, 41). The authors remarked that myotoxicity was correlated with increased concentrations of and exposure time to bupivacaine. The occurrence of myotoxicity can be explained by the fact that liposomal bupivacaine uses relatively high bupivacaine concentrations and sustained release leads to increased exposure time.

Clinically, effects of muscle damage appear negligible except in muscles of the eye, where temporary diplopia after local anesthesia has been reported (42). Continuous infusion of LAs after rotator cuff surgical repair did not lead to worse clinical outcomes. The authors concluded that myotoxicity may be reversible or is not severe enough to affect tissue healing and postoperative outcomes (43). In orthopedic surgery about 0.14% of patients experienced clinical symptoms of LA-induced myotoxicity, which included significant loss of muscle contraction force and tenderness of the operated extremity. Recovery times ranged from 4 days up to 1 year (26). However, clinical symptoms of muscle damage after LA infiltration are prone to be under-reported. Pain and dysfunction after injections administered for post-surgical analgesia can easily be attributed to- or masked by postsurgical pain (33). Furthermore, a variable degree of damage to muscle (and the associated elevated serum creatine phosphokinase levels) will be caused by muscle dissection during the surgical intervention, complicating isolation of myotoxicity induced specifically by bupivacaine.

## Effects of Bupivacaine on Articular Cartilage

Bupivacaine ranks amongst the most chondrotoxic LAs (44). Levobupivacaine (the S-enantiomer of bupivacaine) induces similar chondrotoxicity when compared to racemic bupivacaine, with some studies also reporting increased *in vitro* chondrocyte mortality after 1 h of exposure to levobupivacaine, when compared to the racemic mixture (45, 46). The cytotoxicity of LAs might be due to lipophilicity rather than stereoisomers, possibly explaining these differences (47). The *in vitro* chondrotoxicity has been thoroughly reviewed and is dependent on exposure time, dose and concentration, and occurs at clinically used concentrations (44, 48–50). Interestingly, osteoarthritic joints appear more susceptible to LA-induced damage compared with joints with healthy cartilage. Management of osteoarthritis-associated pain with LA injections can, therefore, lead to increased cartilage damage, potentially resulting in more pain (48). Exposure of cultured chondrocytes to LAs in *in vitro* studies is often limited to 1 h (48). As the elimination half-life of bupivacaine is ~2.7 h, the limited *in vitro* exposure time could hamper translatability of laboratory findings to the clinic (24).

The chondrotoxic effects of bupivacaine solutions and liposomal bupivacaine were studied in a porcine model. The bupivacaine solution (0.5% w/v) was injected in the stifle joint, leading to significant chondrocyte death *in vivo* (33% non-viable cells), when assessing full-thickness cartilage biopsies with live/dead staining 1 week after administration (51). The use of liposomal bupivacaine formulation (1.3% w/v) resulted in a higher chondrocyte viability when compared to bupivacaine HCl (6.2% non-viable cells for liposomal bupivacaine vs. 33% non-viable cells for bupivacaine HCl) and was therefore considered safer for intra-articular use. However, histological changes regarding surface integrity, fibrillation and chondrocyte viability did not show significant differences between bupivacaine solutions and liposomal bupivacaine formulation 1 week after injection in a porcine model. The synovial membrane was not assessed (51).

In another study, the long-term effects of bupivacaine during 48 h of continuous infusion were studied in a rabbit shoulder model (52). No macroscopic or radiological differences were observed between infused glenohumeral joints and controls after 3 months. Cartilage metabolism assessed with sulfate uptake increased after bupivacaine infusion, potentially indicating a regenerative response. No difference in cell density, percentage of live cells, macroscopic or radiographic changes were observed. Therefore, in the model used, articular cartilage has the potential to recover from chondrotoxic effects induced by bupivacaine. However, other animal studies conclude that chondrocyte homeostasis does not fully recover following intra-articular bupivacaine administration. Rats received a 0.5% bupivacaine injection into the stifle joint. The contralateral joint received 0.9% saline with the same volume. Cartilage was assessed histologically after 1, 4, 12 weeks, and 6 months. Bupivacaine did not lead to damage to the chondral surface, or superficial chondrocyte viability up to 12 weeks after injection. After 6 months, chondrocyte density had significantly decreased when compared with the control joint, despite cell viability remaining constant (53).

Clinically, chondrolysis following a single intra-articular LA administration has seldom been reported. This phenomenon is characterized by increasing pain and stiffness in the treated joint, with radiological signs of cartilage breakdown and reduced joint space width. A 2015 systematic review discussed reports of four clinical cases. The rate of chondrolysis when studying continuous infusion was considerably higher compared with single injections, and 97.7% (163 out of 167) of chondrolysis cases reported occurred after continuous infusion and the majority of cases occurred in the glenohumeral joint. It should be noted that in most studies adrenaline was co-administered with bupivacaine (54).

## Effects of Bupivacaine on the Intervertebral Disc

Bupivacaine is a commonly used LA for both diagnosis and treatment of discogenic back pain. However, various *in vitro* studies have shown toxic effects of bupivacaine on intervertebral disc (IVD) cells. Bupivacaine appears to lead to both concentration- and exposure time-dependent necrosis and apoptosis of IVD cells, and decreased matrix synthesis (55–60).

As with osteoarthritic synovial joints, attempts to relieve pain from degenerative disc disease using LAs could lead to an increase of symptoms due to the cytotoxic effects of these LAs. Interestingly, these findings only partially correlate with data from *in vivo* and clinical studies. For instance, increased rates of apoptosis were observed seven days after bupivacaine (0.5%) injection into intervertebral discs of a rabbit model when compared to saline controls, which are in agreement with previous *in vitro* studies (61, 62). However, no significant differences in IVD degeneration as scored on MRI, histological scoring using safranin-O staining, or amount of viable IVD cells between saline and bupivacaine injection were observed after 6 and 12 months. The main cause of damage appeared to be insertion of the needle into the annulus fibrosus (61). When comparing bupivacaine-treated (discography or disc-block, both single injection) IVDs with control discs 5 years after treatment, no significant radiological differences were observed between groups regarding disc height, pain, disability scores or range of motion (63). Accelerated disc degeneration 10 years after discography has been reported, but this study did not specify the injection fluid used (64). Therefore, the observed degeneration following diagnostic or therapeutic disc injections appears to be induced mainly by damage caused by insertion of the needle and/or by the use of a contrast agent (61, 65). A potential explanation for the discrepancies between *in vitro* and *in vivo* or clinical observations might be cell proliferation reported *in vivo* after bupivacaine injection into the IVD, which is potentially associated with a regenerative response (66).

## Effects of Bupivacaine on Bone Repair/Regeneration

Bone (re)generation and formation are essential processes in the recovery period following musculoskeletal interventions. Impaired bone healing can have severe clinical consequences, such as mal- or non-union of fractures or reduced osteointegration of implants. Lucchinetti et al. reported a dose-dependent reduction of mineralized matrix deposition by MSCs during LA exposure, with bupivacaine being the most inhibitory. At bupivacaine concentrations of 250 uM (=0.008%) no osteogenesis was observed *in vitro* (67). These findings are in contrast to studies in animal models assessing bone healing in the presence of bupivacaine. Fourteen and thirty-five days after hematoma infiltration with 0.25% bupivacaine following a closed diaphysis fracture in rat femora, no difference in callus composition, bone tensile strength, or histological appearance was observed between bupivacaine-treated and control groups (68, 69). Similar results were reported in dogs for 0.5% bupivacaine infiltration (70). Clinical studies describing effects of bupivacaine on bone healing is limited. Results of the few studies available seem to agree with findings from the animal studies mentioned. A retrospective cohort study assessing feasibility of hematoma block following femoral fracture in 35 children did not report delayed bone healing (71). The effect of liposomal bupivacaine (120 mg) on bone healing was assessed on X-ray images taken 4–6 weeks after bunionectomy. None of the 158 patients showed evidence of impaired bone healing. However, whether the surgery included any correction osteotomy was not specified. In the case of bunionectomy only, no bone fragments would have to fuse (72).

## Effects of Bupivacaine on Wound Healing

Wound healing is an important regenerative process following surgery. Various drugs are known to be associated with delayed or disturbed wound healing (73). In an *in vitro* wound healing scratch assay, MSCs were cultured using medium supplemented with 20 ng/mL TNF-alpha and exposed to bupivacaine 100 uM (=0.00325%). Following 3 and 6 h of bupivacaine exposure, delayed migration of MSCs and delayed repopulation of the *in vitro* defect size compared with controls were observed (67). Population doubling time increased significantly from 40 to 93 h at these concentrations. At higher concentrations of bupivacaine, a decrease in cell count was observed. This phenomenon was accompanied by LDH release, a marker for cytotoxicity and increased membrane permeability (67).

However, in a rat model of wound healing, no differences regarding amount of collagen fibers, wound tensile strength, and inflammatory parameters were observed between the intervention and control group after 14 days, indicating that bupivacaine did not negatively affect wound healing (74). Similar studies have been performed in mice (75). After 3 days, no differences regarding wound surface, re-epithelialization, or neutrophil numbers were observed compared to controls in both the healthy and impaired healing groups. In contrast to results from the rat model, a non-significant trend toward decreased collagen accumulation in wounds following LA administration was found in mice. However, this study did not objectify wound tensile strength and used a shorter follow-up period (3 vs. 14 days) (74, 75).

Furthermore, results of clinical studies showed no significant differences in wound healing or scar formation in later phases when using liposome bupivacaine or conventional bupivacaine HCl. Overall, satisfactory wound healing in both interventional groups was reported across various surgical models (76). A randomized clinical trial reported no wound healing complications of bupivacaine infiltration compared to control group in human breast surgery (77).

## Effects of Bupivacaine on Neural Tissue

The musculoskeletal system, especially the spinal column, is in close relationship with the central and peripheral nervous system. To obtain anesthesia during orthopedic interventions, administering regional or spinal blocks with bupivacaine are common in the perioperative pain management.

Bupivacaine can lead to significant neuronal cell death *in vitro*. In experiment using human SH-SY5Y neuroblastoma cells, a concentration-dependent decrease in cell viability after exposure to various LAs was observed in 10 min. Bupivacaine had the highest toxic potency of all LAs included in the study. Cell death was primarily due to necrosis, but bupivacaine also led to apoptosis when concentration or exposure time increased (78). These results are in agreement with other reports (79).

*In vivo*, the effect of intraneural injection of both plain bupivacaine solution and liposomal bupivacaine was studied at clinically used concentrations in a porcine model. No persistent neurological deficits were observed 12 h after treatment. The sciatic nerve was excised 2 weeks after injection and assessed for histological nerve injury. Immune cell count, cytokine mRNA and axonal density did not differ significantly in both groups when compared to controls (80). In rabbits, bupivacaine led to moderate neurotoxicity (regarding CSF glutamate concentrations and vacuolation of the dorsal funiculus) of the spinal cord 1 week after intrathecal administration. However, the LA concentrations used in the study were higher than those used clinically (81).

From a clinical perspective, persisting neurological damage from LAs is rare, with risks of neurological injury after *peripheral* nerve blocks estimated to be around 0.04% (82). The risk of lasting damage is lower in *central* nerve blocks (i.e., spinal and epidural applications), with estimates between 1 and 4 in 100.000 cases (83). The incidence of anesthesia-related neurologic complications varies, however, both over time and between studies (83, 84). For example, neurological deficits have been reported in 3–5% of patients 2 weeks after undergoing a brachial plexus block. After 4 weeks, 0.4% of patients still experienced deficits (85). These numbers, apart from being at risk for publication bias, might overestimate the actual damage done by LAs, as injection pressure, needle trauma and patient positioning during surgery can also account for (persisting) neurological damage (83).

## Recent Developments

To both extend duration of action and minimize toxicity, alternative formulations of bupivacaine have been developed. Examples are liposomal formulations and biodegradable polymer matrices, with liposome bupivacaine being the only formulation to have reached market approval presently (10–14). A hydrogel formulations of bupivacaine, liposome bupivacaine, and a conventional bupivacaine solution have been tested *in vivo* in direct comparison (14). Following plantar incision, rats were administered 0.1 mL near the sciatic nerve of either liposome bupivacaine (13.3 mg/mL), hydrogel matrix containing bupivacaine HCl (105 mg/mL), or the hydrogel matrix without any bupivacaine. An irritant rank score was calculated based on histological analysis, grading the presence of inflammatory cells, fibroblasts, neovascularization, fibrosis, necrosis, hemorrhage, and tissue ingrowth into the material. Mechanical pain sensitivity threshold was tested using Von Frey ligaments. Rats treated with the bupivacaine-containing hydrogel matrix tolerated higher mechanical forces on the injured paw compared to the liposomal bupivacaine group and the empty hydrogel matrix group. However, this group also displayed the highest irritant ranking scores after 5 (moderate score) and 42 (slight score) days. The empty hydrogel matrix also displayed moderate and slight irritant rank scores at 5 and 42 days, respectively, indicating local toxic effects of the matrix itself. It is pointed out that only the cumulative score was reported, preventing evaluation of specific local effects on inflammation, vascularization, necrosis and other factors. Furthermore, not only the concentration but also the cumulative dose of bupivacaine differed between the intervention and control group, hampering their comparability. No clinical trials of the hydrogel bupivacaine formulation are available yet. Liposomal bupivacaine formulations have been tested clinically; however, the analgesic advantages compared with plain bupivacaine solutions appear limited (86). *In vivo*, the myotoxic effects of liposomal bupivacaine (13.3 mg/mL bupivacaine HCl) were comparable to 5 mg/mL plain bupivacaine HCl solution after 5 days, and significantly less than a 13.3 mg/mL plain bupivacaine HCl solution. However, the degree of inflammation after 2 weeks was comparable between the 13.3 mg/mL bupivacaine HCl solution and liposomal bupivacaine (41).

## Conclusions and Outlook

Bupivacaine is extensively used in (surgical) treatment of musculoskeletal diseases since its discovery in 1957. In bone regeneration, muscle repair, nerve damage, wound healing, and intervertebral disc damage, the *in vitro* effects of bupivacaine point toward an inhibitory effect, even at concentrations lower and exposure times shorter than those used clinically. Racemic mixtures of bupivacaine enantiomers and levobupivacaine lead to similar degrees of *in vitro* toxicity. However, in both animal and clinical studies, these effects are rarely reproduced and, if they are, appear largely reversible. In IVD, neural and muscle applications, the local toxic effects of bupivacaine displayed reversibility, albeit delayed compared to controls. Clinically observed adverse events following local toxicity of bupivacaine have seldom been reported. The local toxic effects of bupivacaine, when used as perioperative anesthetic, may have minimal impact compared to the extensive tissue damage and systemic response elicited by the surgery itself. Bupivacaine seems to induce chondrotoxicity *in vitro* and up to a certain extent also in *in vivo* and in clinical studies. Therefore, the use of bupivacaine in synovial joints might best be avoided. Recently developed liposomal and polymer matrix formulations of bupivacaine provide a longer duration of action, but with similar degrees of local toxicity. This leaves an unmet need for a LA-formulation that increases the duration of pain blockage without increasing local toxicity. As described in this review, the *in vitro* toxic effects of bupivacaine are rare in *in vivo* studies. Interestingly, if adverse effects are observed *in vivo* or clinically, they appear to be reversible. A possible explanation for the discrepancies observed between *in vitro* and *in vivo* data might be the models used to study *in vitro* toxicity of bupivacaine. Indeed, most of the *in vitro* tests are performed in two-dimensional cell cultures, which are not representative of the complex architecture and systemic absorption and metabolism of the human body. Furthermore, the inflammatory, high-perfusion, regenerative phase taking place after both bupivacaine injection and surgery is overlooked *in vitro*. In summary, this review revealed that current literature report low levels of bupivacaine local toxicity in clinically used concentrations for the majority of musculoskeletal applications.

## Author Contributions

JS and J-JV conceptualized the review. JS performed the literature search and data analysis. SP and JM drafted and critically revised the work. All authors contributed to the article and approved the submitted version.

## Conflict of Interest

The authors declare that the research was conducted in the absence of any commercial or financial relationships that could be construed as a potential conflict of interest.

## Publisher's Note

All claims expressed in this article are solely those of the authors and do not necessarily represent those of their affiliated organizations, or those of the publisher, the editors and the reviewers. Any product that may be evaluated in this article, or claim that may be made by its manufacturer, is not guaranteed or endorsed by the publisher.
