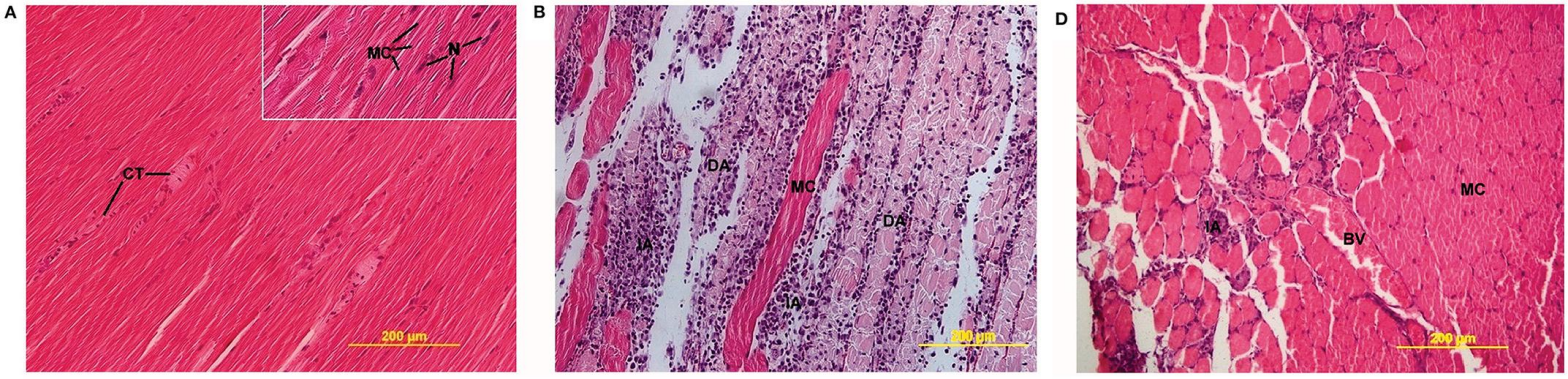# Correction: Comparison of *in vitro* and *in vivo* toxicity of bupivacaine in musculoskeletal applications

**DOI:** 10.3389/fpain.2026.1857584

**Published:** 2026-05-26

**Authors:** Jasper G. Steverink, Susanna Piluso, Jos Malda, Jorrit-Jan Verlaan

**Affiliations:** 1 Department of Orthopedics, University Medical Center Utrecht, Utrecht University, Utrecht, Netherlands; 2 Regenerative Medicine Utrecht, Utrecht University, Utrecht, Netherlands; 3 Department of Developmental BioEngineering, Technical Medical Centre, University of Twente, Enschede, Netherlands; 4 Department of Clinical Sciences, Faculty of Veterinary Medicine, Utrecht University, Utrecht, Netherlands

**Keywords:** bone, muscle, orthopedic, regeneration, tissue, wound healing

Error in figure/table.

Wrong content.

There was a mistake in figure [2] as published. This figure was obtained from reference Öz Gergin, Ö., Yıldız, K., Bayram, A., Sencar, L., Coşkun, G., Yay, A., … Polat, S. (2015). Comparison of the Myotoxic Effects of Levobupivacaine, Bupivacaine, and Ropivacaine: An Electron Microscopic Study. Ultrastructural Pathology, 39(3), 169–176. https://doi.org/10.3109/01913123.2015.1014610 The figure contained an unexpected image overlap as flagged on PubPeer. The figure has thus been removed from the article.

The figure caption now reads:

Light microscopy images of LA-induced myotoxicity. (A) Skeletal muscle 2 days after 0.9% saline injection, showing connective tissue (CT) between normal muscle fibers (MC) (20× magnification, insert 40×). (B)Skeletal muscle 2 days after 0.5% bupivacaine injection, showing degenerative (DA) and inflammatory areas (IA) alternated with MC (20× magnification). (D) Skeletal muscle 2 days after 0.5% levobupivacaine injection, showing incidental IA and blood vessels (BV) between MC (20× magnification). Adapted from: Öz Gergin et al. Comparison of the Myotoxic Effects of Levobupivacaine, Bupivacaine, and Ropivacaine: An Electron Microscopic Study. Ultrastructural Pathology, May 2015. Reprinted by permission of the publisher (Taylor & Francis Ltd.) (34).

The original version of this article has been updated.

**Figure 1:**